# Subnitrate of Bismuth in Diarrhœa of Children

**Published:** 1871-04

**Authors:** 


SUBNITRATE OF BISMUTH IN THE DIARRHŒA OF CHILDREN. Dr. Lochner states, in the *Aerzt. Int. Bl.* (No. 37, 1870), that he treated in the hot summer of 1868 fifty cases of diarrhœa in children, and lost only one patient four months old. The dose generally reached sixty grains a day; and most cases were at once benefitted, whilst in some from two to four days were necessary to arrest the complaint. The author thinks that the actual *modus operandi* is not known, and that, therefore, discovery should be attempted by experiment upon animals.—*Lancet.*

